# Characterization and prediction of gambling behavior in adolescents using the COM-B model

**DOI:** 10.1371/journal.pone.0277520

**Published:** 2022-11-28

**Authors:** Álvaro Botella-Guijarro, Daniel Lloret-Irles, José Vicente Segura-Heras, Juan A. Moriano-León

**Affiliations:** 1 Department of Social and Organizational Psychology, Universidad Nacional de Educación a Distancia (UNED), Madrid, Spain; 2 Health Psychology Department, Universidad Miguel Hernández de Elche, Elche, Spain; 3 Operations Research Center, Universidad Miguel Hernández de Elche, Elche, Spain; Yale University, UNITED STATES

## Abstract

Gambling is an international phenomenon, posing a serious threat to adolescents who begin gambling at a young age. This study aims, to explore gambling behavior in adolescents and interpret its risk factors. We conducted a three-waves cohort longitudinal study assessing gambling and associated risk factors in south-eastern Spain. Data were analyzed using the Capabilities, Opportunities, Motivations, Behavior (COM-B) model and the partial least squares path modelling (PLS-PM) technique. Gambling was measured by frequency and money spent; associated factors were knowledge about gambling, parental attitude towards gambling, risk perception, normative perception, and intention to gamble. These items were assigned as indicators of each construct of the COM-B model—capability, opportunity, motivation, and behavior—using the theoretical domains framework. Once the behavior was performed, feedback on future capability, opportunity, and motivation was observed. Results show that capability, determined by past experience, and opportunity, determined by parental attitudes, motivates adolescents to seek gambling experiences in the future. Identifying such factors that affect gambling behavior in adolescents and establishing relationships between them through a robust theoretical model is essential for designing effective interventions.

## Introduction

One in five European teenagers are involved in gambling. According to reports from the European School Survey Project on Alcohol and Other Drugs (ESPAD), the proportion of 15–16-year-olds who have gambled has changed in recent years, from 14% having gambled at least once in their lifetime in 2015 to 22% having gambled in the last 12 months in 2019. Although the measure has changed between the two waves of data, and interpretation should be made with caution, we can conclude that gambling has become more prevalent among the European population aged 15 to 16. In addition, the ESPAD report classifies 15% as excessive gamblers and 5% as problem gamblers. The classifications of excessive gambler is based on the Consumption Screen for Problem Gambling (CSPG) scale, adapted from Rockloff (2012), and those of problem gambler on the Lie/Bet Questionnaire, adapted from Johnson (1997) [1, 2]. Studies on gambling behavior during the COVID-19 pandemic’s mobility restrictions in Spain showed a reduction in gambling in venues and an increase in online gambling among the population under 25 years of age [3]. In a survey conducted by the Australian Gambling Research Centre during COVID-19, men aged 18–34 were found to be most likely to sign up for online accounts, increase their frequency and monthly expenditure on gambling, and face a higher risk of gambling-related harm, according to the classification provided by the Problem Gambling Severity Index (PGSI), a proxy measure for gambling disorder [4]. Based on estimates by the National Opinion Research Center DSM Screen for Gambling Problems NODS and DSM-IV-MR-J screen, several authors have argued that gambling is a public health issue because of the increase in cases of gambling disorder especially among adolescents [5, 6].

The onset of gambling at an early age increases the risk of developing problem gambling in adulthood, according to the categorization obtained by the PGSI [7–9]. Therefore, it is important to develop interventions aimed at adolescents and young adults that reduce or prevent gambling attitudes. Identifying the factors that affect gambling behavior and establishing relationships between them through a robust theoretical model is essential for designing effective interventions for the prevention of problem gambling in adolescents [10].

There is grounded evidence that identifies factors related to the onset and maintenance of gambling among adolescents. On the individual level, low risk perception has been associated with a higher frequency and intensity of gambling [11, 12]. While adolescents understand that gambling involves risk, they do not see themselves as potential victims [13]. Another individual factor that predicts gambling intention and behavior is perceived control, based on cognitive biases where an individual believes that it is possible to control chance [14, 15]. Once initiated, gambling behavior has a high continuation rate [11, 16, 17]. At the family level, there is strong evidence linking parental attitudes and behavior towards gambling with higher frequency of offspring gambling [18–20]. Similarly, poor, or negligent parental supervision is a common variable for many risky behaviors, including gambling [21].

From a micro-social perspective [22], the normative perception, understood as the belief that gambling behavior is normal in adolescence, maintains a directly proportional relationship with the frequency of gambling [23, 24].

**Fig 1:**
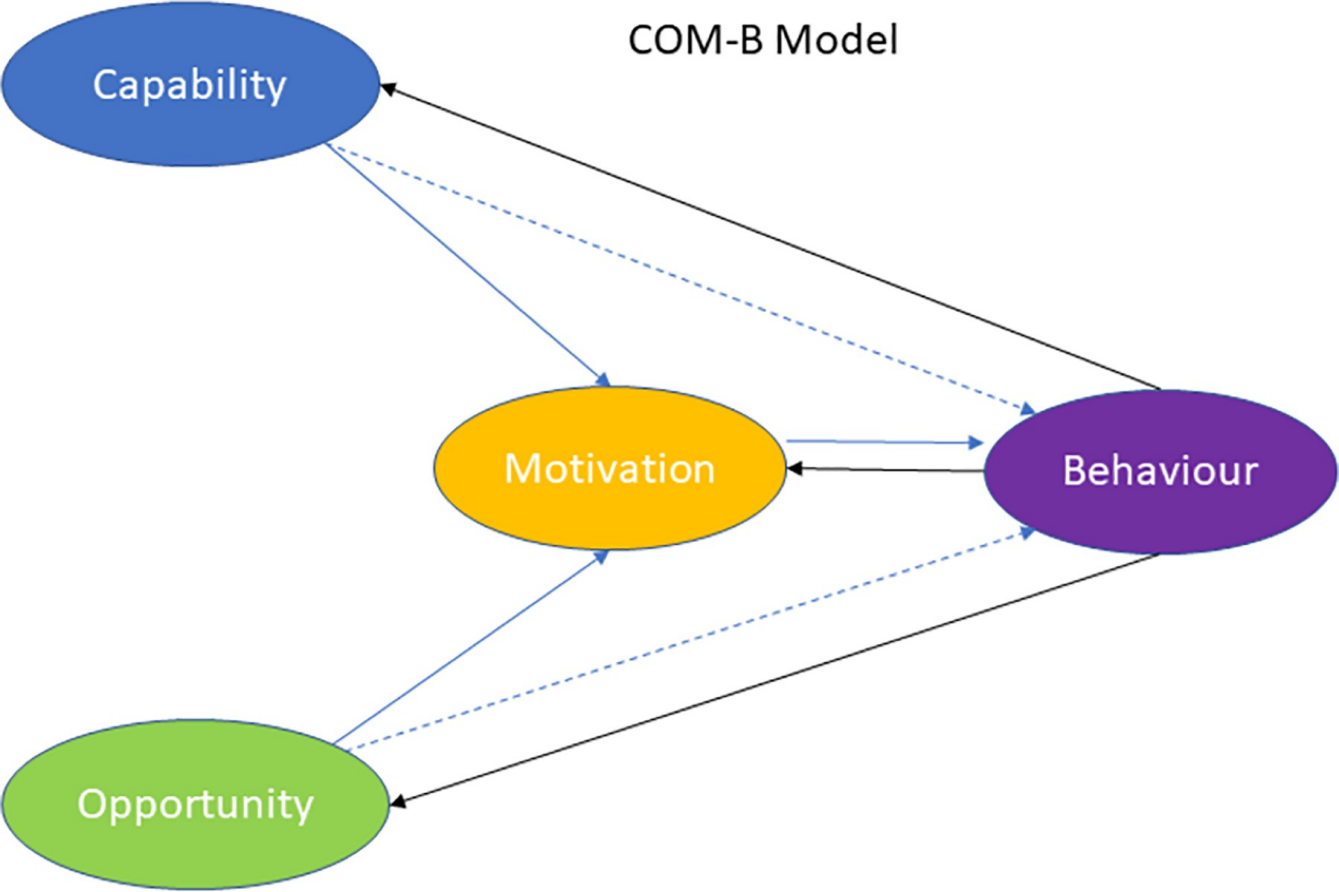
COM-B model. Adapted from West & Michie [28].

Risk factors acquire an even greater significance when they are integrated into a theoretical model. In this regard, the above factors are represented in the *Capabilities*, *Opportunities*, *Motivations*, *Behavior* (COM-B) model [25, 26], which postulates a general framework for behavioral change. The model predicts behavior by combining three parameters (see Fig 1)—*capability* (psychological or physical capability, for example, knowledge and skills), *opportunity* (physical and social environment, factors external to the individual), and *motivation* (reflexive and automatic cognitive processes)—that interact to generate *behavior*. *Motivation* also serves as a central mediator between *capability*, *opportunity*, and *behavior*, as both *capability* and *opportunity* influence *motivation*. Therefore, *capability* and *opportunity* affect *behavior* through direct and indirect pathways [25–27]. West and Michie [28] reformulated the model in such a way that *capability* and *opportunity* act on the relationship between *motivation* and *behavior* as ‘logic gates’ and that, at a given point in time, both *capability* and *opportunity* must be open for *motivation* to be expressed in *behavior*. Ultimately, *behavior* feeds back into *capability*, *opportunity*, and *motivation*. This feedback can create both positive and negative cycles, increasing or decreasing, respectively, the likelihood of the behavior occurring again [28]. Therefore, the model is structured temporally, on three phases: a first phase in which *capability*, *opportunity*, and *motivation* are measured; a second phase in which the execution of the behavior is measured; and a third phase in which *capability*, *opportunity*, and *motivation* are measured, and the possible feedback of the model is assessed.

Given the large number of existing theories of behavioral change and the numerous derived constructs, an integrative framework was designed to synthesize the various key theoretical constructs–the theoretical domains framework (TDF). The TDF synthesizes 33 theories and 128 theoretical constructs on behavioral change into 14 constructs [25, 29]. The framework serves as an aid for assigning each risk factor to each of the constructs of the COM-B model.

This model can help clarify gambling behavior in context [25, 27], diagnose which variables need to change to achieve the desired behavior, and inform the design of interventions for the prevention of abusive gambling in adolescents.

In the field of addictive behaviors, the COM-B model has been shown to be useful for understanding the changes needed to prevent the consumption of alcohol [30, 31] or tobacco [32, 33]. Based on these results, we consider that the model can also be applied to other potentially addictive behaviors. As far as gambling is concerned, to our knowledge, no study has used the COM-B model to analyze predictors of gambling behavior among adolescents. The only reference we have been able to find is McMahon et al. [34], who used the model to implement changes in a harm reduction intervention for gambling behavior in adults.

Considering this evidence, the COM-B model is a promising framework for explaining potentially addictive behaviors. Therefore, the aim of the present study is to understand gambling behavior in adolescents and interpret risk factors according to the COM-B model. To apply the sequential logic of the model in its three measurement phases, we used a longitudinal study with three waves of data on gambling behavior.

The data obtained through the COM-B model was analyzed through partial least squares path modelling (PLS-PM), a technique that allows us to analyze relationships between groups of variables.

This data analysis approach has not been widely used in the study of the COM-B model or the prevention of gambling behavior in adolescents; therefore, it is a novel contribution of the present work. Some examples of the use of PLS and structural equations (SEM) can be found in Howlett et al. [35], applied to physical activity, and in Zou et al. [36], related to self-care in patients with chronic heart failure. The application of the model in the field of adolescent gambling behavior is another novel aspect of this research.

## Methods

### Participants

The sample was recruited in three consecutive years (2016-2017-2018) from 15 randomly selected public educational centers in south-eastern Spain. Seven of the educational centers are in municipalities with less than 50,000 inhabitants and eight with more than 50,000 inhabitants. The main economic activities of these municipalities are agriculture (four centers), industry (five centers) and service-tourism sector (six centers).

In the first measurement (T1) the sample consisted of 2716 students in 9th, 10th, and 11th grades, with a mean age of 15.12 years (SD = 1.03. Range 13–17) and 49.9% female. At the second measurement (T2), 2430 students from 10th, 11th, and 12th grades participated, mean age 16.07 (SD = 0.99. Range 14–19), 54.8% female. At the third measurement (T3), the sample consisted of 1726 students in 11th and 12th grades, with a mean age of 16.52 (SD = 0.76. Range 15–20), 54.0% female. The matched sample of the three points in time– T1-T2-T3 –was composed of 391 students, out of which 55.12% were girls. During the follow-up, there were sample losses (with respect to T1,65.72% in T1 and 85.60% in T2) due to: (1) the impossibility of measuring pupils who changed schools or municipalities in the following academic year, after completing compulsory education; (2) failure to obtain parental authorization and/or absenteeism; and (3) errors or falsification in the anonymous coding of the participants.

Cases aged between 14 and 16 years inclusive, at the time of measurement T1, were selected for the present study, resulting in a sample of 354 cases, 52.54% female. Of these cases, 20.34% have gambled once in the year at T2 (2.82% female), and 4.24% are at-risk or problem gamblers (0.28% female), according to the categorization obtained by the South Oaks Gambling Scale-RA (SOGS-RA).

### Procedure

After obtaining authorization from the competent educational authority, the study was approved by the Ethics Committee of the Miguel Hernández University (DPS.DLI.01.17). First, 30 schools were identified, 9 of which declined to participate for various reasons. Of the remaining, 15 were chosen at random, meeting the criterion of representation of all the regions (2 schools per region). In regions with more than two centers, the schools were selected at random. All the required classes within each school were included. All measures were integrated into a single questionnaire. Each of the students present in the classroom, from the selected schools and courses, completed the questionnaire. In each session, an expert collaborator from the research team distributed the questionnaire and clarified any doubts and questions. The sessions were supervised by a teacher designated by the center. Informed consent was obtained from the parents or guardians of the participants. Adolescents participated voluntarily after being informed of the purpose of the study. The duration of each session was 25–30 minutes.

### Measures

The COM-B model consists of four constructs: *capability*, *opportunity*, *motivation*, and *behavior*. The measurement items for each component of the model were selected as follows. *Capability* was assessed by measuring the frequency of gambling in the last 12 months. It was derived from training or practice, within the theoretical domain “Skills” as proposed in the Theoretical Domains Framework [25, 29]. This training or practice was understood in terms of frequency of gambling in the last year, as the history of past gambling behavior is shown to be a risk factor for current gambling behavior [8, 11, 16, 17, 37]. The more you have played in the last year, the more *capability* to play you acquire. The frequency variable was measured through four items collected and adapted from the European School Survey Project on Alcohol and other Drugs questionnaire (ESPAD), which asked about the number of times each of the five gambling modalities had been used: online sports betting, in-room sports betting, in-room slot machines, online poker or casino games, and in-room roulette. Another theoretical domain related to the *capability* component of the COM-B model is “Knowledge”. This domain was assessed through four items of the accessibility (AC) subscale of the Early Detection Gambling Addiction Risk-Adolescents (EDGAR-A) battery, (Cronbach’s alpha = 0.74) [38], which informs us about the degree of knowledge about where and how to gamble.

*Opportunity* consisted of seven items from the Parental Attitude (PA) subscale–parental attitude towards gambling–of the EDGAR-A battery, with a Cronbach’s alpha representing internal consistency of .86. The higher the score, the more favorable the parents were towards gambling.

*Motivation* was constructed using 13 Likert-type items selected from the EDGAR-A subscales: seven items from risk perception (Cronbach’s alpha = 0.79), three items from normative perception (Cronbach’s alpha = 0.75), and three items from intention to gamble (Cronbach’s alpha = .84).

*Gambling behavior* included two items: the maximum amount of money gambled in the last 12 months, with 7 answering options (from 0 = *€0* to 6 = *more than €70*); and the score on the South Oaks Gambling Scale-RA (SOGS-RA) [39, 40], composed of 12 dichotomous items and one Likert-type item (Cronbach’s alpha = 0.73). Based on frequently used cutoff scores for the SOGS-RA, non-problem gambling was defined as scores of 0 and 1, at-risk individuals had scores of 2 and 3, and problem gambling was defined as scores of 4 or above [41].

The variables related to the *capability*, *opportunity*, and *motivation* constructs were assessed at time T1 and T3, while those related to gambling behavior were measured at time T2.

### Statistical analysis

The partial least squares (PLS) technique was employed to obtain estimations and fit indices for the proposed model in the context of the structural equation model (SEM) using R software [42], SEMinR package, version 2.1.0 [43]. Before running the analysis, the data distributions were verified for anomalies such as outliers, missing values, and deviation from normality.

The relationships between the groups of variables are established by considering the theory, or prior knowledge, regarding the phenomenon under analysis. Furthermore, we assumed that each group of variables plays the role of a theoretical concept represented in the form of a construct or latent variable [44–46]. The PLS-PM analysis also allows for more flexibility in the assumptions on the distribution of data and the minimum sample size.

This technique allows for a choice between the formative and the reflective measures of the constructs. In the formative measures, each item represents a dimension of the construct’s meaning; thus, removing an item signifies that the construct loses some of its meaning. In the reflective measures, items represent manifestations of the construct and are competitive with each other. In the present study, we opted for a formative model of measurement for all constructs [47, 48], in which the measurement items “cause”, or are antecedents of, the theoretical constructs. Hair, J.R. et al. [49] combine the methodological concepts of this technique in a single text with the use of this R package as a method for estimating PLS path models.

The proposed model was fitted in its entirety, starting from the aforementioned items as the outer model. This was done so that, in the case of the motivation construct, the items comprising the variables risk perception, normative perception, and intention to gamble would form the measurement model of the latent variable motivation, without interposing the former three variables as intermediate latent variables between the items and the constructs of the COM-B model (*capability*, *motivation*, *opportunity*, and *behavior*).

The structural model was also adjusted (inner model, path model between the constructs) to obtain the weight of each relationship. The goodness-of-fit indices were obtained for the mean model and the structural model. The model obtained was evaluated and an alternative model was adjusted following the same procedure.

Hair et al. [44] considered that the evaluations of formative measurement models include three aspects: convergent validity, the evaluation of collinearity issues, as well as the evaluation of the significance and relevance of the indicators. For the evaluation of structural models, it is recommended to use the coefficients of determination (R^2^), the size and significance of the path coefficients, and the effect sizes (f^2^).

The goodness-of-fit indices of the two models were compared.

## Results

A first model was estimated, from which items with collinearity issues (variance inflation factor [VIF] greater than 3.3) were removed, namely, those measuring opportunity OPPT1.4, OPPT1.5, OPPT3.4, and OPPT3.5.

**Fig 2:**
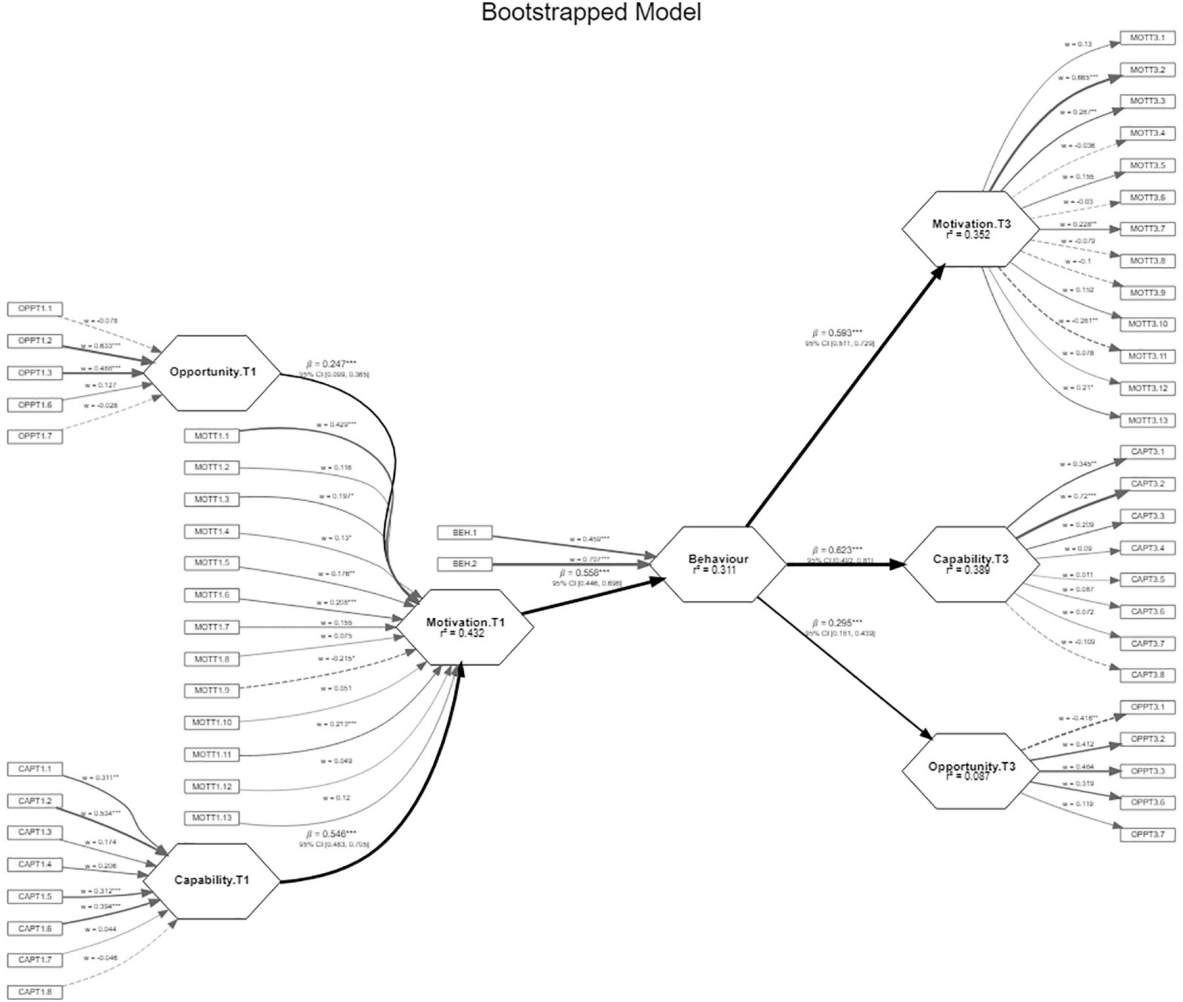
Initial model. The measurement model (rectangles) and the structural model (hexagons) are shown. Factor loadings for each indicator (w), standardized regression coefficients or path coefficients (β) with confidence intervals and coefficients of determination (R^2^) are indicated. The greater the thickness of the arrow, the greater the loading or path coefficient.

Fig 2 shows the resulting structural model containing the algebraic sign, magnitude, and statistical significance of the standardized regression coefficients (path coefficients). Their magnitudes ranged from 0.247 to 0.558, all being statistically significant, based on the bootstrapping process performed.

The R^2^ obtained was generally moderate, except for the one associated with *opportunity* at T3, which was weak. *Capability* and *opportunity* at T1 explained 54.6% and 24.7% of *motivation* at T1, respectively. *Motivation* at T1 explained 55.8% of *behavior* at T2, the latter explaining 59.3% of *motivation*, 62.3% of *capability*, and 29.5% of *opportunity* at T3.

Cohen’s f^2^ assesses whether the omitted construct has a substantive impact on the endogenous constructs: 0.02 would be associated with a small effect; 0.15 with a medium effect; and 0.35 with a large effect. In our case, opportunity had a small effect on motivation at T1, as did *behavior* on *motivation* at T3.

**Fig 3:**
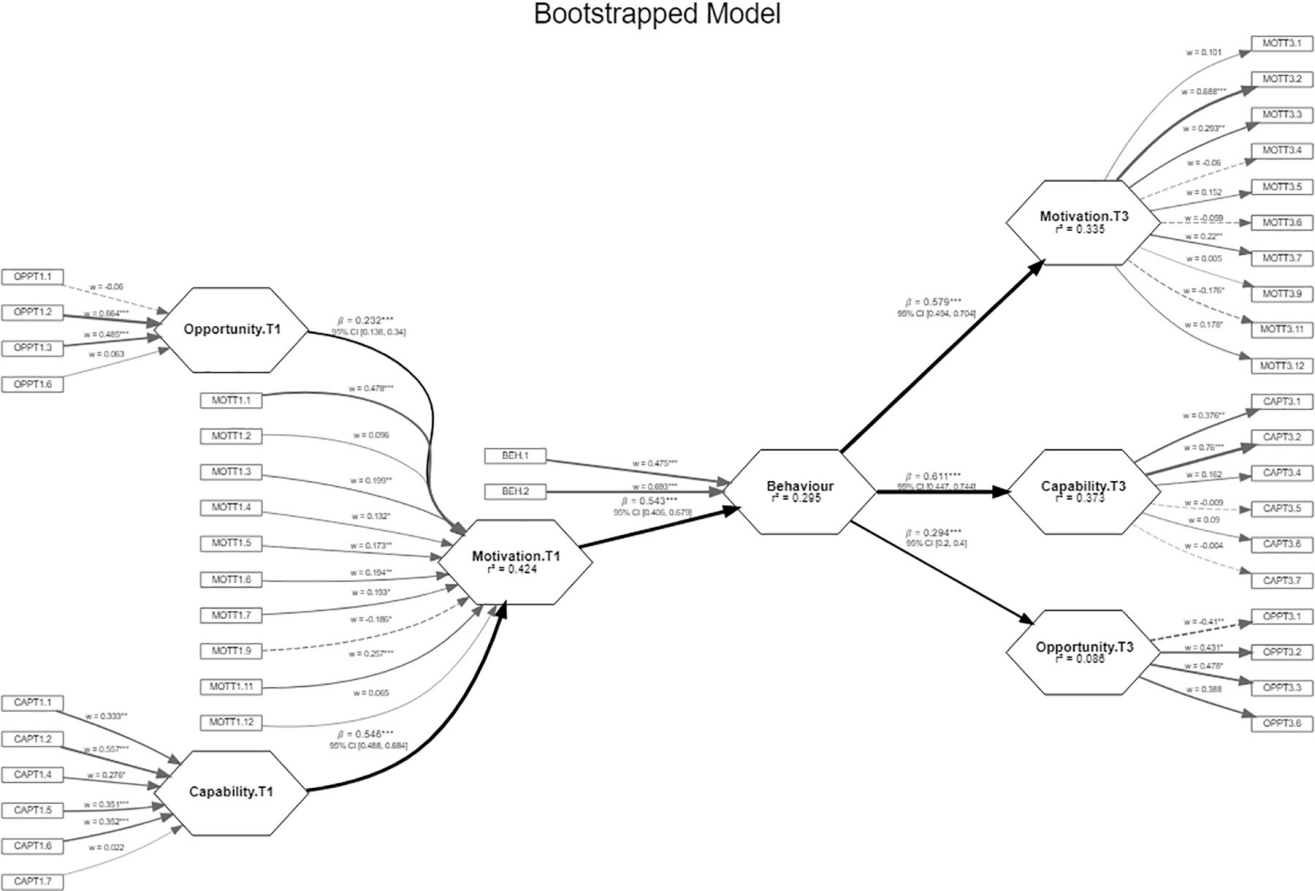
Adjusted model. The measurement model (rectangles) and the structural model (hexagons) are shown. Factor loadings for each indicator (w), standardized regression coefficients or path coefficients (β) with confidence intervals and coefficients of determination (R^2^) are indicated. The greater the thickness of the arrow, the greater the loading or path coefficient.

A more adjusted model was tested, eliminating those measurement items with non-significant weights, without the model losing quality. The adjusted model is shown in Fig 3.

The items OPPT1.7, OPPT3.7, CAPT1.3, CAPT1.8, CAPT3.3, CAPT3.8, MOTT1.8, MOTT1.10, MOTT1.13, MOTT3.8, MOTT3.10 and MOTT3.13 were removed; thus, we obtained a more parsimonious model without losing the quality of fit in the structural model. The evaluation indices of the two models are listed in Tables 1 and 2.

**Table 1:** Determination coefficient (R^2^) y Coef. path. Initial model (adjusted model).

	Mot.T1	Behavior	Mot.T3	Cap.T3	Opp.T3
R^2^	0.432 (0.424)	0.311 (0.295)	0.352 (0.335)	0.389 (0.373)	0.087 (0.086)
Adj. R^2^	0.429 (0.421)	0.309 (0.293)	0.350 (0.334)	0.387 (0.372)	0.085 (0.084)
Capability.T1	0.546 (0.546)				
Opportunity.T1	0.247 (0.232)				
Motivation.T1		0.558 (0.543)			
Behavior			0.593 (0.579)	0.623 (0.611)	0.295 (0.294)

Note: R^2^ is the determination coefficient; Adj. R^2^, the adjusted determination coefficient. For Motivation in T1 (Mot. T1), Behavior, Motivation in T3 (Mot.T3), Capability in T3 (Cap.T3) and Opportunity in T3 (Opp.T3). Without parentheses: initial model, in parentheses: adjusted model. The rest are path coefficients between correlated constructs.

**Table 2:** Cohen’s f^2^. Initial model (Adjusted model).

	Mot.T1	Behavior	Mot.T3	Cap.T3	Opp.T3
Capability.T1	0.413 (0.414)				
Opportunity.T1	0.080 (0.072)				
Motivation.T1		0.452 (0.418)			
Behavior			0.543 (0.505)	0.636 (0.596)	0.095 (0.095)

Note: Cohen’s f^2^ for Motivation in T1 (Mot. T1), Behavior, Motivation in T3 (Mot.T3), Capability in T3 (Cap.T3) and Opportunity in T3 (Opp.T3). Without parentheses: initial model, in parentheses: adjusted model.

## Discussion

The aim of the present study was to understand gambling behavior in adolescents and to interpret risk factors according to the COM-B model. For this purpose, a sample of adolescents was observed for three consecutive years with respect to their gambling behavior and other related variables.

As they continue gambling, their capability will increase, they will possibly be more motivated to gamble again (*motivation*) and think that their parents have a favorable attitude towards gambling behavior (*opportunity*) and other related variables.

The results showed that *capability*, understood as knowledge and experience about gambling, is a predictor of motivation to gamble, and consequently, of gambling. These findings are consistent with previous studies where experience leads to gamble in a significant proportion of adolescents [17, 50–52]. Marketing strategists in the service of the gaming industry know that the likelihood of gambling increases with prior experience. A worrying approach is the marketing strategy of offering simulated gambling scenarios that reproduces “real” gambling activities, which enables the users to gamble without investing money. Simulated gambling in social networks or on Internet gaming platforms, fosters the onset of gambling and promotes an increasing worry regarding adolescents [53, 54]. Therefore, gamble experience, or *capability*, acts in a self-feeding spiral, in which more gambling experiences will increase their *capability* and, possibly, *motivation* to gamble again.

The *opportunity* parameter of the COM-B model, referring to a social or physical environment which encourages gambling, was represented by the favorable attitude of parents toward gambling in our study. The results establish that *motivation* to gamble increases when parents do not convey an anti-gambling attitude, with a significant but small effect. These findings are supported by previous studies, where home characteristics, particularly parental attitude, are linked to a higher likelihood of gambling among their offspring [55].

Although the questionnaire used was not expressly designed to use the COM-B model, the results obtained were acceptable. For example, St-Pierre et al. [56] found that a model based on an extension of the Theory of Planned Behavior (TPB) using SEM explained 29.8% of the variance in the frequency of gambling in the last three months and 28.5% of the intention to gamble. Other studies found that the Theory of Reasoned Action only predicted small amounts of variance in gambling intentions [57]. In this sense, the application of the COM-B model and the PLS-SEM technique to adolescent gambling is a novelty that improves the explanatory capacity of the indicators used, since this model model predicts behavioral performance related to the circumstances, that is, the constructs of *capability*, *motivation*, and *opportunity*, that may control it in the future.

The *capability* construct has the greatest explanatory weight for *motivation*, which can be understood as a reflection of the importance of past experiences, as well as autonomous actions, that strengthen intrinsic motivation in terms of intentions. *Capability* is derived from Bandura’s concept of self-efficacy. It can also be regarded from the perspective of the Theory of Planned Behavior (TPB) which includes “perceived behavioral control” referring to the conviction that one can successfully gamble and attain a desired goal.

The construct *opportunity* had little weight in the estimated model. This may be due to the lack of important indicators for the formation of the construct, which are detailed in the limitations of the study below.

While the longitudinal design provides robustness to the study, 12 months may be a long period to examine the effects of *capability*, *motivation*, and *opportunity* on gambling behavior. Given the formative nature of construct development, it would be advisable to carefully select the most appropriate indicators of each construct for a particular intervention study and justify their selection based on sound explanations supported by empirical evidence. For example, in this study, the construct *opportunity* suffered from the absence of indicators related to accessibility or peer group gambling behavior, which are important factors in explaining adolescent gambling [17, 51, 58]. And that the relationships between *capability* and *behavior* are biased, since the frequency of play has been included as a measure of the *capability* construct, because of the possible improvement in the skins to gambling, but this variable is still gambling behavior (the result) and, consequently, produces biases in the relationships between *capability* and *behavior*. In this sense, it is advisable to use qualitative techniques and follow the tools proposed for the design of interventions with the COM-B model [25, 27]. In general, results should be interpreted considering the limitations of self-report and cohort studies, including potential biases and participant attrition between measurements [17, p. 14]. In addition, it would have been desirable to include children who were not in the regular education system. Although they are a minority, their unique socio-demographic characteristics would enrich the results. In this sense, it should be considered that the sample loss of the study may have left out a certain adolescent population group, which partly reduces the generalizability of the results. Finally, the drawbacks of the concentrated location of the sample must be considered, and caution is required when generalizing the results.

Our results support the use of the COM-B Model as a framework to predict adolescent gambling behavior. The weight of *capability* in gambling motivation proves that those variables should be considered in preventive interventions that aim to delay the onset of early gambling experiences and thus reduce the likelihood of more severe gambling behavior.

Considering the ages of the participants in the study, it is suggested that prevention of gambling should start before the age of onset. The worrying prevalence of gambling among adolescents highlights the need for evidence-based preventive educational interventions. Future research should examine the interaction between indicators related to *opportunity*, that is, accessibility or peer group gambling behavior that could predict adolescent gambling behavior.
